# An examination of the epidemiological features of hand, foot, and mouth disease in a Gansu Province city between 2014 and 2024

**DOI:** 10.3389/fpubh.2026.1777089

**Published:** 2026-04-10

**Authors:** Sheng Li, Yinfan Gou, Rongxuan Zhang, Guoping Xi, Di Yang, Jinyu Wang

**Affiliations:** 1 Lanzhou Second People's Hospital, Lanzhou, China; 2 School of Public Health, Gansu University of Chinese Medicine, Lanzhou, China; 3 Pingliang Centre for Disease Control and Prevention, Pingliang, China; 4 Lanzhou First People's Hospital, Lanzhou, China; 5 School of Public Health, Lanzhou University, Lanzhou, China

**Keywords:** chi-square test, epidemiological characteristics, hand, foot and mouth disease, Pingliang city, public health policy

## Abstract

**Objective:**

To examine the epidemiological features of hand, foot, and mouth disease (HFMD) in Pingliang City, Gansu Province, between 2014 and 2024 in order to provide guidance for developing, putting into practice, and improving HFMD preventive and control policies and measures in Pingliang City.

**Methods:**

Methods of descriptive epidemiology were used. From January 1, 2014 to December 31, 2024, data on HFMD cases in Pingliang City, Gansu Province, as well as yearly demographic data from every county and district were gathered. Excel and SPSS 27.0 software were used for data organization and *χ*^2^ testing.

**Results:**

From 2014 to 2024, a cumulative total of 4,641 HFMD cases were reported in Pingliang City, with an average annual incidence rate of 22.53 per 100,000 population. The highest incidence rate was recorded in 2017 (39.18 per 100,000), while the lowest occurred in 2022 (2.91 per 100,000). The incidence rate exhibited an overall upward trend, with statistically significant differences (*χ*^2^ = 1560.248, *p* < 0.001). The male-to-female ratio was 1.52:1, with males exhibiting a higher incidence rate (27.09 per 100,000) than females (17.96 per 100,000), a difference statistically significant (*χ*^2^ = 190.609, *p* < 0.001). The age distribution was concentrated among children aged five years and below (4,079 cases), accounting for 87.89% of all reported cases, with the highest proportion occurring among one-year-olds (1,442 cases, 31.07%). Occupational distribution primarily involved children living in scattered households, totalling 3,116 cases (67.14%). Pingliang City reported cases monthly, with a seasonal peak occurring between May and July. Regional distribution revealed the highest incidence rate in Jingchuan County (42.64 per 100,000), while Zhuanglang County recorded the lowest (14.81 per 100,000). Differences in HFMD incidence rates across counties were statistically significant (*χ*^2^ = 772.442, *p* < 0.001).

**Conclusion:**

Children under five years of age living in scattered households remain a high-risk group for HFMD. Targeted preventive measures should be implemented for key populations, including public awareness campaigns, health education initiatives, and the promotion of vaccination to reduce incidence rates.

## 1 Introduction

Hand-foot-mouth disease (HFMD) is a globally prevalent enteric infectious disease (1), predominantly affecting children aged five years and below, characterised by high contagiousness and seasonal outbreaks (2). The disease is primarily caused by enterovirus 71 (EV 71), Coxsackievirus A16, or other enterovirus serotypes (3). Clinical manifestations predominantly include fever, oral vesicles, and rash on the hands, feet, and buttocks. A minority of cases (particularly those involving EV-A17 infection) may progress to severe illness, presenting with neurological symptoms and cardiopulmonary failure, potentially leading to fatality and posing a significant threat to children’s health. HFMD transmission occurs through multiple routes, including close contact, droplet transmission, and indirect contact with contaminated surfaces, having caused outbreaks in numerous regions.

Since 2008, China has classified HFMD as a Category C notifiable infectious disease. Its incidence and disease burden remain persistently high across multiple surveillance systems, establishing it as a significant infectious threat to paediatric health in China (4, 5). Gansu Province, situated in northwestern China, is one of the regions with high HFMD prevalence, exhibiting fluctuating upward trends in reported incidence in recent years (6). Located in eastern Gansu, Pingliang City’s geographical environment, climatic conditions, population structure, and healthcare resource distribution may influence the epidemiological characteristics of HFMD. Currently, systematic epidemiological research on the medium-to-long-term trends in Pingliang remains limited. Therefore, this study conducts a systematic analysis of the epidemiological characteristics of HFMD in Pingliang City, Gansu Province, from 2014 to 2024, aiming to provide a reference basis for further improving the prevention and control strategies for HFMD in Pingliang.

## 2 Data and methods

### 2.1 Data sources

#### 2.1.1 Case data

Data on hand, foot and mouth disease cases in Pingliang City from 1 January 2014 to 31 December 2024 were sourced from the ‘Infectious Disease Surveillance System’ within the ‘China Disease Prevention and Control Information System’. When extracting cases, the screening criteria were set to include reviewed clinical diagnoses and confirmed cases, with ‘hand, foot and mouth disease’ selected as the disease type. The data was exported in January 2025 to ensure that all revised reports were included. The case information collected included date of onset, date of diagnosis, gender, age, occupational classification and current address.

#### 2.1.2 Population data

The data on the permanent resident population of Pingliang City and its counties and districts, broken down by gender and age group for the period 2014–2024, are sourced from the annual 《Pingliang City Statistical Yearbook》 and the statistical bulletins of the respective counties and districts. The population data for the counties and districts are used to calculate regional incidence rates.

### 2.2 Case diagnostic criteria

The diagnosis and classification of hand, foot and mouth disease are based on the ‘Guidelines for the Diagnosis and Treatment of Hand, Foot and Mouth Disease (2018 Edition)’ and the ‘Diagnostic Criteria for Hand, Foot and Mouth Disease (WS 588-2018)’, both of which are industry standards of the People’s Republic of China (7).

### 2.3 Research methods

#### 2.3.1 Data collation and quality control

Data organization was carried out using Excel 2019. Quality control measures included: (1) logical verification: screening for and reviewing outliers in the database; (2) handling of duplicate reports: in cases where the same patient was reported multiple times, duplicate records were identified based on name, date of birth and guardian’s contact details, with only the first instance of the illness being retained; (3) data cleansing: removing case data where the address was unknown or the patient was not a local resident.

#### 2.3.2 Descriptive epidemiological analysis

Using descriptive epidemiological analysis methods, we examined surveillance data on hand, foot and mouth disease (HFMD) in Pingliang City from 2014 to 2024. Specific analyses included: (1) Epidemiological overview: Calculating annual reported HFMD cases and incidence rates to describe overall epidemic intensity; (2) Demographic characteristics: Analysing case distribution by gender, age group, and occupational setting (e.g., community-dwelling children, nursery attendees, school pupils); (3) Temporal distribution: Monthly incidence counts were tabulated, with incidence curves plotted to identify seasonal peaks; (4) Regional distribution: Annual average reported incidence rates were calculated for each district and county within Pingliang City, with comparisons made regarding regional incidence disparities. Given this study’s reliance on routine surveillance data, analysis focused on the aforementioned core epidemiological characteristics. Other potentially influential factors (such as pathogen typing or proportion of severe cases) were excluded from this analysis due to limitations in data availability.

#### 2.3.3 Statistical analysis

Statistical analysis was performed using SPSS 27.0 software. The chi-square test was employed to determine whether differences existed in incidence rates across different years, genders, and counties/districts. The significance level was set at *α* = 0.05, with *p* < 0.05 indicating statistically significant differences.

## 3 Results

### 3.1 Epidemiological overview

From 2014 to 2024, Pingliang City reported a cumulative total of 4,641 cases of hand, foot and mouth disease, with an average annual incidence rate of 22.53 per 100,000 population. The highest number of cases was reported in 2017 (751 cases), with an incidence rate of 39.18 per 100,000 population. The lowest number of cases was reported in 2022 (53 cases), with an incidence rate of 2.91 per 100,000 population. The incidence rates across different years showed statistically significant differences (*χ*^2^ = 1560.248, *p* < 0.001), as shown in Table 1.

**Table 1:** Number of reported cases and reported incidence rate of hand, foot and mouth disease in Pingliang city, 2014–2024.

Annual	Number of reported cases (cases)	Population (in 10,000)	Reported incidence rate (per 100,000)
2014	325	197.71	16.44
2015	713	195.31	36.51
2016	368	193.31	19.04
2017	751	191.69	39.18
2018	609	189.38	32.16
2019	405	187.17	21.64
2020	72	184.49	3.90
2021	127	182.47	6.96
2022	53	182.25	2.91
2023	540	178.58	30.24
2024	678	177.27	38.25
Total	4,641	2,059.63	22.53

### 3.2 Demographic characteristics

From 2014 to 2024, among the cumulative reported cases of hand, foot and mouth disease in Pingliang City, 2,796 cases (60.25%) were male and 1,845 cases (39.75%) were female, yielding a male-to-female ratio of 1.52:1. The reported incidence rate was 27.09 per 100,000 for males and 17.96 per 100,000 for females, with a statistically significant difference (*χ*^2^ = 190.609, *p* < 0.001), as shown in Table 2. The age distribution of HFMD cases was predominantly concentrated in children aged 5 years and below, accounting for 4,079 cumulative reported cases (87.89% of all reported cases). Among these, the highest proportion was observed in 1-year-olds, with 1,442 cases (31.07%); Regarding occupational groups, children living in scattered households constituted the largest cohort with 3,116 cases (67.14%), followed by nursery-aged children with 1,093 cases (23.55%), schoolchildren with 385 cases (8.30%), and other occupational groups with 47 cases (1.01%), as detailed in Table 2.

**Table 2:** Demographic characteristics of hand, foot, and mouth disease in Pingliang city, 2014–2024.

Project	Number of reported cases (cases)	Composition ratio (%)	Reported incidence rate (per 100,000)
Gender			
Male	2,796	60.25	27.09
Female	1,845	39.75	17.96
Age (years)			
0	344	7.41	
1	1,442	31.07	
2	799	17.22	
3	635	13.68	
4	543	11.70	
5	316	6.81	
6	169	3.64	
≥7	393	8.47	
Occupation			
Children of the diaspora	3,116	67.14	
Infant and toddler care children	1,093	23.55	
Student	385	8.30	
Others	47	1.01	

### 3.3 Temporal distribution

**Figure 1:**
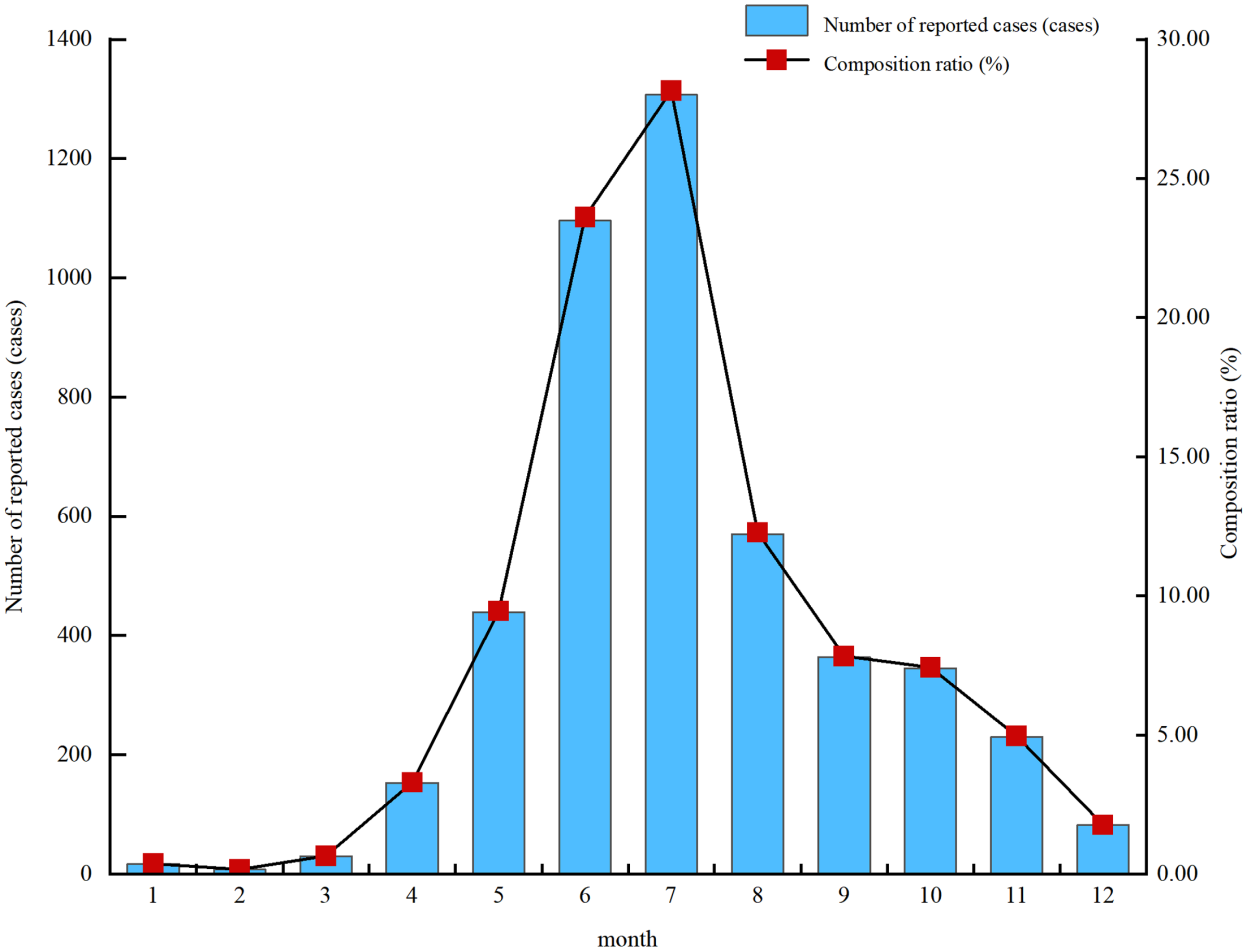
Temporal distribution of reported hand, foot and mouth disease cases in Pingliang city, 2014–2024 [cases (%)].

From 2014 to 2024, Pingliang City reported cases every month, exhibiting distinct seasonal patterns. Incidence peaked during summer months, with case numbers increasing from April onwards and reaching their highest between May and July. The majority of cases occurred between May and August, while February recorded the lowest number at 8 cases (0.17%). This distribution followed a unimodal pattern, as shown in Figure 1.

### 3.4 Regional distribution

**Figure 2:**
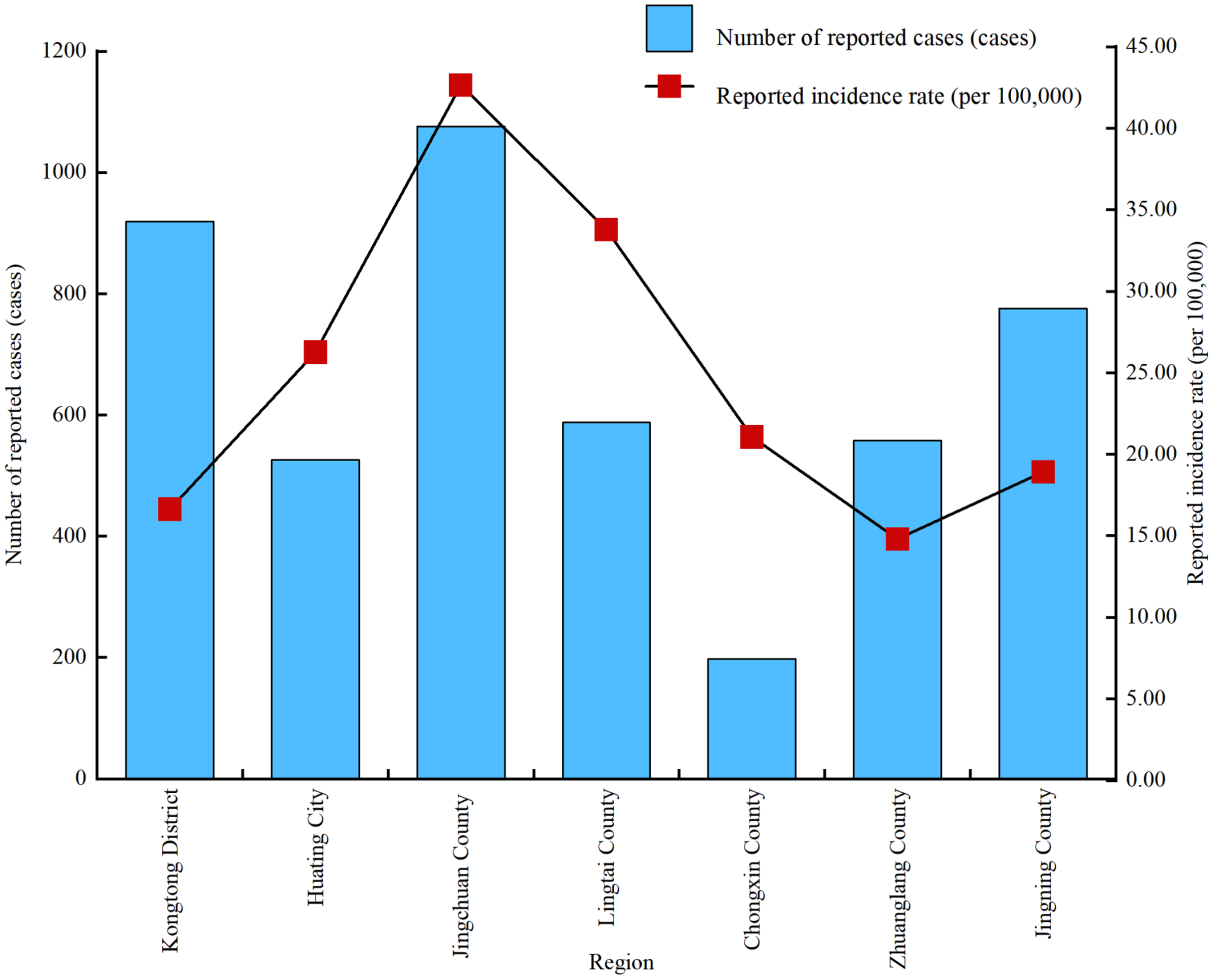
Reported incidence of hand, foot and mouth disease in counties and districts of Pingliang city, 2014–2024.

From 2014 to 2024, all counties and districts within Pingliang City reported cases of hand, foot and mouth disease. Among these, Jingchuan County (1,076 cases), Kongtong District (919 cases), and Jingning County (776 cases) ranked as the top three in cumulative reported cases, accounting for 59.71% of the total. Jingchuan County (42.46 per 100,000), Lingtai County (33.79 per 100,000), and Huating City (26.26 per 100,000) recorded the three highest annual average incidence rates of HFMD. The incidence rates varied significantly across counties (*χ*^2^ = 772.442, *p* < 0.001), as shown in Figure 2.

## 4 Discussion

The findings of this investigation reveal that between 2014 and 2024, Pingliang City reported a cumulative total of 4,641 cases, exhibiting marked annual fluctuations with an overall upward trend. The average annual incidence rate stood at 22.53 per 100,000 population, lower than the national average reported incidence rate for hand, foot and mouth disease (HFMD) of 134.59 per 100,000 population documented by Zhang Jing et al. (8). The significantly higher number of reported HFMD cases in 2017 compared to other years may be attributed to the relatively low incidence in 2016, which led to an accumulation of susceptible individuals. This created conditions for an outbreak in 2017, resulting in the phenomenon of ‘immunity debt’ (9). From 2020 to 2022, Pingliang City exhibited a marked downward trend in reported HFMD incidence. Analysis suggests this may relate to non-pharmaceutical interventions implemented during the COVID-19 pandemic, which reduced opportunities for infectious disease transmission (10). Since the onset of the COVID-19 pandemic in 2020, enhanced disease prevention awareness among residents, coupled with reduced participation in group gatherings due to control measures and public education campaigns, effectively diminished HFMD transmission risks. With the implementation of Category B management for COVID-19, the resumption of social activities and increased population mobility led to a rebound in reported incidence rates from 2023 onwards. This phenomenon aligns with the findings of Zhang Yutong et al. (11), highlighting the need to balance societal recovery with disease surveillance responsiveness in infectious disease control. Vigilance is required regarding the resurgence of infection risks associated with the phenomenon of ‘immunity debt’ (9, 12).

In this study, the incidence of hand, foot and mouth disease was significantly higher among males than females, consistent with the findings of Yang Jianjun et al. regarding the investigation of hand, foot and mouth disease in Gansu Province from 2010 to 2021 (13). Analysis suggests this may be attributable to boys’ poorer hygiene practices, their tendency toward greater physical activity and preference for outdoor pursuits, thereby increasing exposure to pathogens and enteroviruses (14, 15). Additionally, research indicates that females generally exhibit superior immune responses to various infections compared to males, which may also contribute to the higher incidence among males (16). Regarding age and occupational distribution, the high-incidence group for HFMD primarily comprises scattered children aged 0–5 years, with the highest incidence observed among one-year-olds, consistent with relevant research findings (17, 18). This suggests that the decline of maternal antibodies in infants, coupled with the immaturity of their own immune systems, constitutes the primary cause of their susceptibility. We should prioritise scattered children aged 0–5 years, developing targeted intervention measures and health education activities. Particular attention should be given to children aged 0–3 years, actively fostering good hand hygiene and behavioural habits. Knowledge on preventing and controlling HFMD should be disseminated through multiple channels to enhance parental disease awareness, enabling early detection, isolation, and treatment to reduce the incidence of HFMD (19).

The temporal distribution indicates that the seasonal peak of hand, foot and mouth disease in Pingliang City occurs during summer (May–August), consistent with previous research findings (20). This aligns with the ‘single-peak’ distribution pattern observed in most northern regions such as Beijing, Liaoning, and Heilongjiang (18). Relevant studies suggest that climatic variations including temperature, humidity, and sunlight exposure influence both the proliferation and survival duration of the disease pathogen (21, 22). Summer heatwaves and increased population density due to school reopenings heighten transmission risks. This underscores the need for enhanced early warning systems and prevention guidance for childcare facilities, communities, and healthcare institutions during peak summer transmission periods (23).

Hand, foot and mouth disease is distributed across all counties and districts of Pingliang City, but the reported incidence in Jingchuan County is particularly prominent, forming a distinct regional cluster. Investigations reveal Jingchuan County is predominantly agricultural, potentially linked to its high proportion of rural residents, relatively weak sanitation infrastructure, poor living environments and hygiene conditions, and frequent population gatherings (24). This suggests that HFMD epidemics may be influenced by multiple factors including environmental conditions, school distribution, and population activities. It is necessary to conduct more in-depth investigations into environmental and behavioural risk factors in this region and implement targeted interventions, such as improving drinking water sanitation, strengthening supervision of childcare facilities, and promoting centralised vaccination, to reduce infection risks in high-incidence areas (25).

In summary, the prevention and control of hand, foot and mouth disease in Pingliang City should prioritise scattered children under five years of age. Since 2016, an inactivated EV71 vaccine has been available in China, recommended for children aged 6 months to 5 years. This vaccine has been demonstrated to effectively reduce EV71-associated HFMD cases and severe complications (26). Therefore, in addition to non-pharmaceutical interventions, we recommend that local health authorities in Pingliang City actively promote EV71 vaccination. Particularly in high-incidence areas such as Jingchuan County and among high-risk groups like scattered children under five years of age. Increasing vaccination coverage, combined with health education and environmental sanitation improvements, can significantly alleviate the burden of HFMD in this region. Strengthening surveillance and response during peak seasons and in high-incidence areas, alongside advancing comprehensive measures through multi-departmental collaboration including health education, environmental improvements, and immunoprophylaxis can effectively reduce HFMD incidence and disease burden, thereby promoting public health.

## Data Availability

The original contributions presented in the study are included in the article/supplementary material, further inquiries can be directed to the corresponding authors.
